# Pigmented Epithelioid Melanocytomas and Their Mimics; Focus on Their Novel Molecular Findings

**DOI:** 10.3390/biology10121290

**Published:** 2021-12-08

**Authors:** Erol C. Bayraktar, George Jour

**Affiliations:** 1 Department of Pathology, NYU Grossman School of Medicine, New York, NY 10016, USA; erol.bayraktar@nyulangone.org; 2 The Ronald O. Perelman Department of Dermatology, NYU Grossman School of Medicine, New York, NY 10016, USA; 3 Interdisciplinary Melanoma Program, NYU Grossman School of Medicine, New York, NY 10016, USA

**Keywords:** pigmented epithelioid melanocytoma, whole-genome methylation array, melanoma

## Abstract

**Simple Summary:**

Pigmented epithelioid melanocytoma (PEM) is a rare entity with a controversial biological behavior. Some of these tumors behave in an indolent manners while others can locally spread. Herein, we review the clinical presentations, the pathological features as well as the genomic signatures associated with this rare entity. We also report an example of a challenging case of PEM that we encountered and show how usage of novel molecular diagnostic techniques focusing helps addressing this diagnostic conundrum.

**Abstract:**

Pigmented epithelioid melanocytoma (PEM) is a unique tumor with significantly pigmented appearance and indolent behavior; however, it can demonstrate cytological atypia and metastasize to local lymph nodes. Clinical and histomorphological overlap between PEM and its lower or higher-grade mimics can make it difficult to distinguish in certain cases. Genomic, transcriptomic and epigenetic data indicate that PEMs are molecularly distinct entities from other melanocytic neoplasms and melanomas. In addition, methylation studies are emerging as a tool that can be useful in difficult cases. In this review, we focus on the clinical, histopathologic and recent insights in the molecular features of pigmented epithelioid melanocytic melanocytomas and their mimics. We also present a challenging case that was resolved using methylation analysis providing a proof of concept for using epigenetic studies for similar challenging cases.

## 1. Background

Pigmented epithelioid melanocytoma (PEM) is an intermediate-grade melanocytic neoplasm which was historically reported as animal-type melanoma or equine melanotic disease [1,2], given their clinical appearance similar to tumors arising in horses with typical heavily pigmented appearance. Despite aggressive nomenclature used in the past, such as melanosarcoma, animal-type melanoma, melanophagic melanoma or pigment-synthesizing melanoma [3,4,5,6,7,8,9,10,11,12,13,14,15,16], it was later discovered that these tumors are uniquely lower grade tumors that are unlikely to spread systemically. These tumors were later coined together with epithelioid blue nevi (EBN) of Carney Complex to become PEM, as these two entities were noticed to be histologically indistinguishable [1]. Molecular characterization of these lesions was able to confirm that PEM and EBN are indeed identical, and they stand out differently from commonly compared blue nevi, equine melanomas or classical melanomas [17].

## 2. Clinical Features

PEM has a predilection for younger population but occurs in a wide range of ages (median age, 27 years) with no ethnic susceptibility. The occurrence of these neoplasms is not associated with sun exposure; therefore, it is also seen in genital or mucosal sites and can be seen in populations that are usually less susceptible to sun-induced melanomas such as Hispanic, African–American, Asian and Persian people [1,18]. Most of these tumors arise de novo but occasionally, a dermal or congenital nevus is reported along [17]. PEM most commonly involves extremities, but it is almost reported in most places including back, scalp, ankle, sacrum, genitals, oral mucosa and shoulders [6,19,20]. Clinically, PEM appears as blue-black plaques or nodules greater than 1 cm in diameter and can be present for months to years [21]. Significant pigmentation is often noted at first encounter. Sometimes, an ulceration is present and is more commonly reported in sporadic lesions in comparison with PEM arising in Carney Complex (previously called EBN) [17]. PEM is an intermediate-grade tumor with low probability of systemic spread (3.2%) despite its ability to metastasize to regional lymph nodes (41%) or locally recur (7.9%) [22]. Deaths due to PEMs are very rare and can be associated with additional mutations in metastatic cases, such as in *BRAF V600E*, *TP53* or *PIK3CA*. Although there is no robust data on sentinel lymph node biopsy (SLNB), most of these patients undergo wide excision and SLNB given the uncertain malignant potential. [23].

## 3. Histological Features

PEM tumors have sheets and nodules of heavily pigmented melanocytes in deep dermis with epithelioid and spindled appearance. Epithelioid cells tend to accumulate centrally, whereas spindly cells are present individually or as groups and are more frequently observed closer to the periphery of the lesions [24]. Melanophages make up less than 10% of the cells [1]. Lesions frequently extend into deep dermis or subcutaneous tissue along periadnexal tissue (hair follicles, pilar muscle, eccrine coils, and neurovascular bundles). PEM is composed of 3 cell types: blue nevus such as dendritic cells, polygonal cells with dark pigmentation and epithelioid cells with abundant cytoplasm and large vesicular nuclei [17]. Two distinct populations of epithelioid cells are described. Most frequently seen are medium-sized round to polygonal cells with heavy cytoplasmic pigmentation, forming sheets and nests. The nuclei of these cells are small, vesicular, round to oval in shape and nucleoli are seen to be prominent. It is not clear if these cells are melanocytic in nature since some are reported to have features of tumor infiltrating macrophages. The other population is large cells with abundant cytoplasm, seen somewhat difficult to identify due to irregular contours and they lack pigmentation. They might have dot-like melanin granules at the periphery creating a rim around a halo of non-pigmented cytoplasm, sometimes giving them a fried-egg appearance [1,25,26]. Mitotic activity in PEM is very low (0–3 mitoses/mm^2^) [1]. Features such as regression or vascular invasion are very uncommon in PEM [27]. There is no correlation of surveillance with presence of ulceration, degree of atypia, mitotic activity or lymph node metastasis [1]. Lesions express high levels of MART-1. S-100 and HMB-45 are also expressed but variably [16]. Mi-TF and CD68 immunostaining can also help highlighting the tumor cells [20,28].

## 4. Genomic and Transcriptomic Signatures Associated with PEM

**Figure 1:**
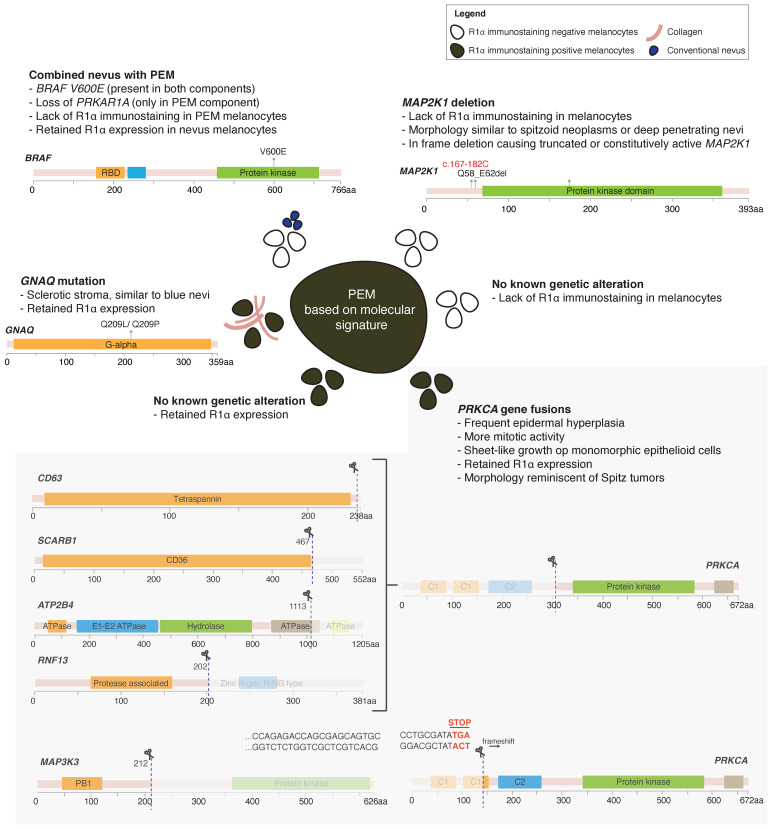
Different molecular subtypes of PEM.

The diagnosis of PEM based on morphology can be challenging. Molecular studies on difficult primary cutaneous melanocytic tumors are recommended using fluorescent in situ hybridization (FISH) or comparative genomic hybridization to establish the diagnosis. The two most common genetic alterations in PEM are fusions in protein kinase C alpha (*PRKCA*) and inactivating mutations in protein kinase cAMP-dependent type I regulatory subunit alpha (*PRKAR1A*), both located in 17q24.2. The *PRKCA* fusion type usually forms solid sheets of monomorphic melanocytes which is more commonly observed in younger patients [29,30,31]. On the other hand, the *PRKAR1A*-mutated lesions have more cytologic heterogeneity and might include a conventional nevus component that has nests separated by collagenous fibrous bands [32]. The *PRKAR1A*-mutated subtype occurs in patients with Carney Complex, whereas both *PRKCA* and *PRKA1A* altered subtypes can be seen sporadically. Based on its molecular phenotype and immunostaining pattern for R1α, six different subtypes are defined for PEM [30] (Figure 1).

### 4.1. PRKAR1A and Its Role in Sporadic and Syndromic PEM

Germline mutations in the gene encoding *PRKAR1A*, a tumor suppressor gene, is associated with Carney Complex phenotype and mutated in 65% of the Carney Complex patients [33]. The *PRKAR1A* gene consists of 11 exons which spans across approximately 21 kb of the genome. The product of this gene is the R1α protein that regulates cyclic AMP and protein kinase A signaling, the major signal transduction pathway required for melanin production. Therefore, loss of R1α expression is consistent with its role in pathogenesis as it helps explain the significant pigmentation in these tumors [34]. Before merging under the umbrella term “PEM”, “epithelioid blue nevi of Carney Complex” and “animal-type melanoma” were two histologically indistinguishable entities [1], therefore begging the question of how molecularly similar these two tumors were. Indeed, genomic studies identified loss of heterozygosity on the locus of *PRKAR1A* gene (chromosome 17q22-24) in sporadic PEMs. This was supported by immunochemistry studies performed for R1α, which is coded by the *PRKAR1A* gene [17]. Loss of R1α reactivity was seen in all the Carney Complex associated PEMs and 82% of sporadic PEMs. It is highly suggested to perform R1α staining for diagnosis for PEMs, as it allows the distinguishing of its potential mimics such as conventional, cellular and malignant blue nevi, deep penetrating nevi and Spitz nevi [17]. However, R1α staining should be interpreted carefully. Unlike Carney Complex-associated PEMs, loss of R1α staining is restricted to melanocytes in sporadic PEMs; whereas reactivity can be seen in epithelioid cells, inflammatory cells and melanophages. *PRKAR1A* mutations are not specific of low-grade PEM per se. In fact, hypercellular lesions (melanocytes outnumbering melanophages) with increased mitotic activity can be concerning for melanoma. In such cases with chromosome number alterations or loss of p16 protein expression, in the absence of TERT promoter mutations, PEM can be regarded as possibly higher-grade [30]. On the other hand, when TERT promoter mutations are present, regardless of *PRKAR1A* status, melanomas should be considered.

### 4.2. PRKCA Role in PEM

Genomic analysis of sporadic PEMs using targeted DNA sequencing and RNA sequencing with a panel of 1700 cancer-related genes demonstrated *PRKCA* fusions to be the most commonly observed genetic event (31%) [31]. In contrast to PEM without *PRKCA* fusion, tumors with this fusion have areas of sheet-like growth of monomorphic epithelioid cells in almost all cases. In addition, prominent epidermal hyperplasia and higher dermal mitotic count is seen. On the other hand, those cases that do not have *PRKCA* fusions are noted to have aggregates of epithelioid to oval-shaped melanocytes separated by fibrotic collagen and melanophages [31]. *PRKCA* is an isoform of protein kinase C and fusions of this gene can lead to constitutive activation of its kinase domain [29]. Although mutations affecting *PRKAR1A* and *MAPK21* are reported in some melanomas, alterations in *PRKCA* are uncommon in malignant melanomas [35,36,37].

### 4.3. Additional Molecular Changes Seen in PEM

Other gene abnormalities can be frequently detected in PEM include *BRAF*, *MAP2K1*, *GNAQ*, *CTNNB1*, *NRAS*, *NF1*, *NTRK1* (Table 1). The *BRAF* mutation is commonly noted in PEMs that are identified as part of a combined nevus. Based on this finding, it is presumed that many PEMs can harbor *BRAF V600E* mutations and in these instances, they arise due to a genetic alteration that leads to *PRKAR1A* inactivation in a conventional or common acquired melanocytic nevus which typically has *BRAF V600E* mutations. Therefore, components of conventional melanocytic nevi can be expected around these lesions. *GNAQ*/*GNA11* mutations are more often seen in blue nevi but can also be in some PEMs [30,31] and when present, these lesions might appear morphologically more similar to blue nevi. *CTNNB1* mutation is more commonly seen in deep penetrating nevi but is also reported in some PEMs. In contrast, PEM typically bear no mutations in *TERT* promoter or unbalanced copy number changes in known oncogenes (*RREB1*, *MYB1*, *CCND1*, *CKND2A*) and presence of these molecular changes should raise the suspicion for melanomas.

## 5. Whole-Genome Methylation and Its Role in Diagnosing PEM

DNA methylation is an essential epigenetic regulatory mechanism in cells, and it is associated with changes in gene expression. Depending on the methylation patterns of DNA or histones, set of genes expressed or repressed can be drastically different. These patterns can result from either somatically acquired changes or reflect the cell of origin. Based on this, methylation profiles of cancers are explored and demonstrated as an extremely useful tool for accurate classification in melanocytic tumors and central nervous system (CNS) tumors [38,39]. In a recent study, a total of 82 CNS tumor methylation classes were identified based on the methylation profiles of 2801 specimens, measuring methylation levels at 27,578 CpG dinucleotides in 14,495 genes using the Illumina Methylation Assays. This method is shown to be very comprehensive, highly robust and reproducible even from small samples and can results in change of diagnosis in up to 12% of prospective cases. In this regard, methylation profiling of melanocytic tumors can be highly valuable, especially in significantly atypical lesions where melanomas are being considered. Some clinical and histomorphological overlap exists between melanomas and PEMs, making it difficult to accurately distinguish these entities in such cases. However, melanocytic schwannomas, melanomas and melanocytomas appear to have distinct characteristics based on their methylation profile [38,39]. Although classification based on the methylation profile of the tumors is tested and proven to be reliable, fast and effective in CNS tumors, application of the same algorithm for accurate diagnosis of cutaneous PEM is warranted and is currently under investigation by our group.

### Case Report

**Figure 2:**
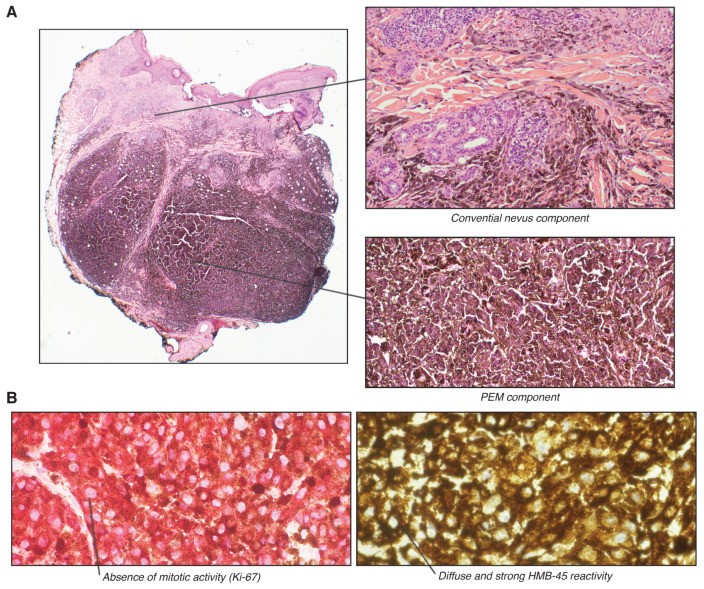
Microscopic examination. (**A**). (left) Low-power view of the tumor, presenting as a 4 mm nodule located in the deep dermis and extending into the subcutaneous fat. (top right) Heavily pigmented melanocytes have epithelioid and spindled appearance. (bottom right) Combined blue nevus-like component. (**B**). (left) Ki-67 immunohistochemistry showing absence of mitotic activity. (right) HMB-45 immunohistochemistry shows diffuse and strong reactivity in tumor cells.

**Figure 3:**
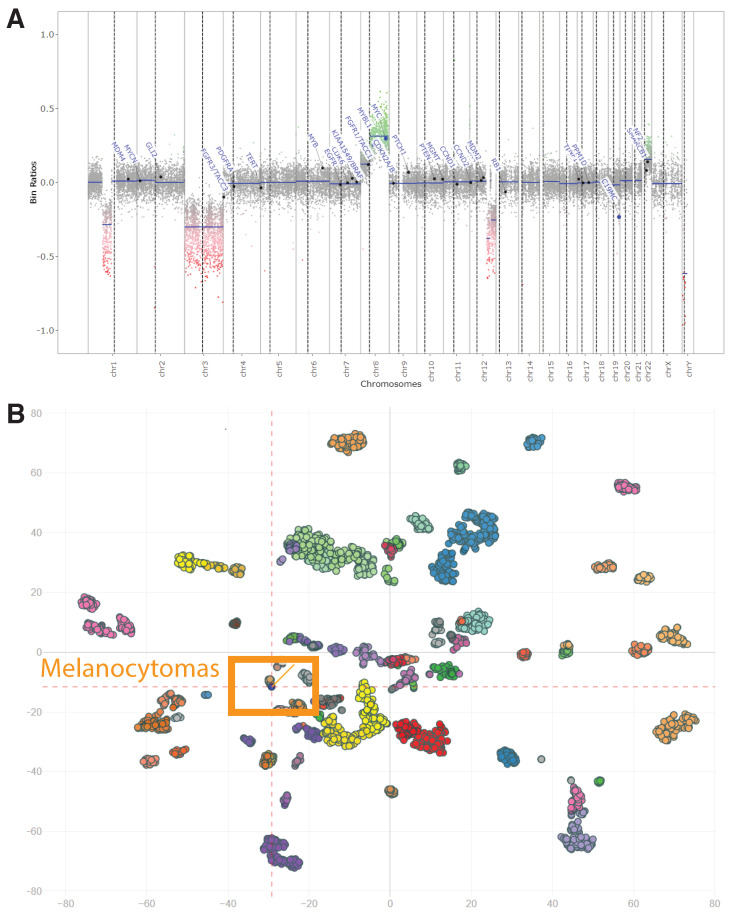
Methylation profiling of the tumor. (**A**). Genome-wide copy number profiles determined from the Illumina Infinium HumanMethylation450 array. The x-axis represents genomic location with each dotted black line marking transition across chromosomes. The y-axis represents signal intensity. Gains represent positive, losses negative deviations from the baseline. Patient tumor demonstrating a relatively stable genome. Please note that there is whole chromosome 3 loss and whole chromosome 8 gain that do not meet the cutoffs for gains (0.5) or losses (−0.5) as per validation of the assay. (**B**). t-SNE visualization of methylation classifier groups. Plot of t-SNE dimensions derived from all methylation sites for all samples in the methylation classifier group and individual points represent each sample. Patient tumor (orange line) is displaying similarity with the melanocytomas group (orange box).

A 34-year-old male presented with a 6 mm bluish nodule, slowly growing on his forehead. An excisional biopsy was performed, which revealed a pigmented lesion with rare mitotic figures and multiple microscopic satellites, extending into fat (Clark level V) to a depth of at least 4 mm (Figure 2). Sentinel lymph node biopsy was negative for neoplasm. Immunohistochemical stains for Melan-A and HMB-45 were diffusely reactive and β-catenin showed non-specific cytoplasmic staining. Ki-67 demonstrated a low proliferative index (<5% in tumor cells). Four-color in situ hybridization was performed to rule out melanoma which showed normal results. Fusion analysis for 104 using targeted RNA sequencing related genes did not reveal any gene rearrangements including *PRKCA* and *PRKAR1A*. Targeted mutation analysis for over 50 cancer-related genes showed *GNA11* c.626A>T p.Q209L oncogenic mutation. Finally, whole-genome DNA methylation profiling and t-Distributed Stochastic Neighbor Embedding (t-SNE) cluster analysis were performed as described above. Genome-wide copy number profiles determined from the DNA methylation data failed to reveal significant copy cumber changes (Figure 3A). t-SNE cluster analysis matched our case to the group of melanocytomas (Figure 3B). Methylation profiling of tumors offers highly efficient and reliable information for classification of tumors and future studies aiming to explore the optimal use of this technique will warrant improved diagnostic and management approaches for pigmented lesions when there is a concern for malignancy.

## 6. Mimics of PEM

Similar to PEMs, there are various other congenital and acquired melanocytic lesions with similar features under the heterogenous group of dermal dendritic proliferations [24,40]. Common to all these lesions are presence of dendritic melanocytes in the dermis. Many of these lesions also have a blue-gray color due to the abundance of melanin pigment combined with the Tyndall effect, in which selective absorption of longer wavelength by dermal melanin and reflection of shorter wavelength from the skin causes the typical bluish appearance. The hypothesized cell of origin for these tumors are melanocytic cells arrested during migration from the neural crest to the skin during embryonic development. Alternatively, it is also suggested that they can be Schwann cells with melanocytic differentiation or another neural crest-derived cell type demonstrating melanocytic or Schwann cell differentiation. Immunohistochemical staining shows invariable HMB-45 positivity in dendritic melanocytes in addition to positivity with the melanocytic markers S-100 and MART-1. To distinguish these entities from one another, clinical, histologic, and molecular features should be considered (Table 2).

## 7. Evaluation of Pigmented Melanocytic Lesions―Where to Draw the Line

Rare melanocytic lesions such as PEM should be referred to specialized centers as they might be quite challenging to diagnose. Histological features such as mitotic counts, pleomorphism and necrosis might not be helpful for intermediate-grade tumors, resulting in intra- and inter-observer diagnostic variability. Furthermore, numerous melanocytic tumors metastasizing to lymph nodes, especially PEMs, can be mistaken for melanomas and therefore should be interpreted carefully. Perhaps, ruling out a melanoma would be one of the most important considerations when evaluating for PEM, as clinical course is almost entirely indolent compared to melanomas. Four-color FISH for *CCDN1*, *MYB*, *RREB1* and *CEP6* has been developed and suggested as a tool for that purpose, screening for common chromosomal changes in melanomas [41,42]. However, sensitivity of this method is limited by possibility of genetic alterations other than these commonly seen chromosomal copy number changes. SNP array is an alternative option that emerged in the recent years that could help addressing FISH shortages but cannot provide comparative classification as the one generated by methylation-based classifier algorithms [43]. Here, we provide a proof of concept that methylation analysis can provide similar data to that seen in SNP array but also help classify borderline melanocytic neoplasms such as PEM. Indeed, methylation profiling provides equal resolution for gene copy number changes but also facilitates powerful data-driven characterization of tumors with their molecular phenotypes in a one-step method. The use of methylation profiling is an emerging approach that has shown promising results in classification of melanocytomas and melanomas in a cohort of CNS tumors. Future studies aiming to validate the utility of this platform in the setting of borderline cutaneous melanocytic neoplasms is needed as it could refine our current diagnostic algorithms.

## Figures and Tables

**Table 1:** Molecular alterations detected in PEM.

Substitutions		Fusions	Indels
*BRAF* V600E	*SMARCA4* R1419H	*SCARB1*- *PRKCA*	*ALK* del.
*BRAF* G469E	NRAS G12C	*CD63*-*PRKCA*	*TERT* promoter del.
*CTNNB1* T41A	*POLE* C2164Y	*ATP2B4*-*PRKCA*	*PRKAR1A* hemizygous del.
*EIF1AX* c.-152C>T	*POLE* V2108M	*MAP3K3*-*PRKCA*	*MAP2K1* c.167-182C del.
*PRKAR1A* c.178-1G>A	*POLE* A1288T	*RNF13*-*PRKCA*	*MAP2K1* I103_K104del
*PRKAR1A* P210L	*TERT* A326V	*TPR*-*NTRK1*	*MAP2K1* Q58_E62del
*FLG* Q1748K	*TERT* E648V	*MYO5A*-*NTRK3*	*PRDM9* W563_S618dup
*FLG* R1758S	*CDK2A* L16fs		*FLG* A3567_Q3568ins
*FLG* R1712	*GNAQ* Q209P		
*FLG* GR3378ET	*GNAQ* Q209L		
*PRDM9* E829D	*RAC1* hotspot missense mutation		
*PRDM9* R842S	*PTCH1* inactivating mutation		
*CACNA1C* E1948K	*TERT* promoter mutations		
*CACNA1C* T330M			

**Table 2:** Summary of the mimics of PEM.

	Clinical Features	Histologic Features	Immunohistochemical Features	Molecular Alterations
Dendritic blue nevus	Well-demarcated papule	Dermal sclerosisDiffuse rather than nested dermal componentNo nuclear pleomorphism	(+) S100, elan-A, HMB-45 (diffuse), SOX10 Low Ki- 67Retained BAP1, p16	GNAQ or GNA11
Cellular blue nevus	Can also involve subcutaneous and soft tissue (muscle, bone)	Nuclei are not epithelioidNo pigmented and clear epithelioid cellsDermal sclerosis	(+) Melan-A, HMB-45 (diffuse), SOX10(+/−) S100, CD34 Low Ki- 67Retained BAP1, p16	GNAQ or GNA11
Deep penetrating nevus	Rarely involves lymph nodes	No dendritic cellsNo mitotic activity	(+) Melan-A, HMB-45 (diffuse), SOX10, Cyclin D1(+) LEF1, β-catenin (nuclear)(+/−) S100, BRAF V600E(−) PRAMELow Ki- 67	BRAF, MAP2K1, CTNNB1
Pigmented epithelioid melanocytoma (PEM)	Sporadic or Carney ComplexCommonly involves lymph nodesNo distant metastasis	Abundant melanophagesEpidermal hyperplasiaInfiltrative bordersUlceration or necrosis rare	(+) Melan-A, HMB-45 (diffuse), SOX10(+/−) S100, BRAF V600E, PRKAR1ARetained p16Low Ki-67	PRKCA, PRKAR1A, GNAQ, MAP2K1
Combined nevus	Combination of variants of acquired nevus, blue nevus, Spitz nevus or PEM	The cellular features of two components need to be architecturally distinct	As per components	As per components
Cutaneous and pilar neurocristic hamartoma	Recurs, can metastasize and be lethal	Nevomelanoctyic, neural and pigmented dendritic cell elements	(+) S100, HMB-45, Melan-A, CD34	
Atypical cellular blue nevus	Similar to blue nevusCan involve lymph nodesCan be large (>10 cm)	Abundant melanophagesFocal cytologic atypiaOccasional mitoses (<2/mm^2^)No atypical mitotic figures or tumor necrosis	(+) Melan-A, HMB-45 (diffuse or patchy), SOX10(+/−) S100, p16 (variable)Intermediate Ki-67	GNAQ, CYSLTR2, PLCB4
Melanoma arising within cellular blue nevus	Similar to blue nevusCommonly involves lymph nodesDistant metastasisPoor prognosis	Abundant melanophagesWidespread necrosisHigh mitotic rate (>2/mm^2^)Atypical mitotic figuresVascular invasionUlceration or necrosis possible	(+) Melan-A, HMB-45 (patchy), SOX10 (+/−) S100, p16 (variable), BAP1High Ki-67	GNAQ, GNA11, PLCB4, CYSLTR2, BAP1, SF3B1, EIF1AX, TERT promoter, chromosomal gains/losses
Melanomas that arise from deep penetrating melanocytoma or DPN	Similar to DPN or deep penetrating melanocytomaCommonly involves lymph nodesDistant metastasis	Abundant pigmentationEpithelioid and spindled cellsSimilar to melanoma	Predicted to be similar to deep penetrating nevus	BRAF, MAP2K1, CTNNB1, TERT promoter, chromosomal gains/losses
Melanoma metastasizing to the skin	Single or multiple nodules	Abundant melanophagesAtypical melanocytesHigh mitotic rate (>2/mm^2^)Stromal reaction	(+) Melan-A, HMB-45 (patchy), SOX10 (+/−) S100, p16 (variable), BAP1, PRAMEHigh Ki-67	TERT promoter, chromosomal gains/losses

## Data Availability

Data available on request from the authors.
